# Commentary: Control of Body Weight by Eating Behavior in Children

**DOI:** 10.3389/fped.2016.00014

**Published:** 2016-03-01

**Authors:** Tony Kuo

**Affiliations:** ^1^Los Angeles County Department of Public Health, Los Angeles, CA, USA

**Keywords:** child obesity, food environment, speed of eating, body weight, public health

Zandian and colleagues’ article on the control of body weight by eating behavior in children draws upon the experience of several disciplines. They reviewed the evidence for the impact of brain physiology, genetics, diet and exercise, and pharmacological interventions in counteracting the worldwide increase in pediatric body weight (1). The authors concluded that a reason for the limited success of these and other weight control interventions in the past has been the misinterpretation of where the locus of control lies – that is, “body weight is mainly under external control” as opposed to the viewpoint that cognition plays a predominant role. This and other conclusions in the article are not new *per se*, as the foundation for addressing “external control” factors is deeply rooted in public-health practice. Indeed, public-health interventions that have focused on policies, systems, and environmental changes in the community have become popular in recent years and have been used extensively by a number of health authorities in developed countries (2). In the United States, for example, growing number of federal initiatives are introducing non-traditional, non-medial strategies to address the rising weight of children. These strategies have included targeted efforts, such as land use policies that support walking in the community; joint use agreements between schools and community entities to increase open space for physical activity; implementation of active transportation policies to improve the walkability of neighborhoods; and mandatory nutrition guidelines in food venues across a range of settings to promote low-energy and low-sugar foods (3). In most cases, these multisector efforts targeted socioecological influences (2) or “external control” factors that Zandian and colleagues alluded to. However, the authors suggested that child obesity shared similarities with the physiological constructs of eating disorders, such as anorexia, and based on these similarities, they concluded that the speed of eating as controlled through computerized feedback technology could be featured in the fight against the obesity epidemic (1, 4). Although intriguing and supported by data from a randomized controlled trial (4), Zandian et al.’s recommendation requires clarification of context and further investigation, as the limited success of the aforementioned diet, exercise, and pharmacological interventions all share common problems – evidence of efficacy and proof-of-concept are available, but research on scale and spread, which affects real world implementation, are not (5). In short, although the authors’ effort to address the “external control” factors that drive the obesity epidemic aligns well with most health authorities’ approach to this public-health problem, their focus on using computerized feedback seems somewhat counterintuitive to their own conclusion. The latter remains focused on cognition and individual health education support, is health systems centric, and may not dramatically improve the actual food environments that influence consumer (parental) choice, especially for those who live in low socioeconomic status areas.

## Author Contribution

The author confirms being the sole contributor of this work and approved it for publication.

## Conflict of Interest Statement

The author declares that the research was conducted in the absence of any commercial or financial relationships that could be construed as a potential conflict of interest.
